# Answering hastily retards learning

**DOI:** 10.1371/journal.pone.0195404

**Published:** 2018-04-25

**Authors:** Yosuke Yawata, Kenichi Makino, Yuji Ikegaya

**Affiliations:** 1 Graduate School of Pharmaceutical Sciences, The University of Tokyo, Tokyo, Japan; 2 Center for Information and Neural Networks, National Institute of Information and Communications Technology, Suita City, Osaka, Japan; Tokai University, JAPAN

## Abstract

Appropriate decisions involve at least two aspects: the speed of the decision and the correctness of the decision. Although a quick and correct decision is generally believed to work favorably, these two aspects may be interdependent in terms of overall task performance. In this study, we scrutinized learning behaviors in an operant task in which rats were required to poke their noses into either of two holes by referring to a light cue. All 22 rats reached the learning criterion, an 80% correct rate, within 4 days of testing, but they were diverse in the number of sessions spent to reach the learning criterion. Individual analyses revealed that the mean latency for responding was negatively correlated with the number of sessions until learning, suggesting that the rats that responded more rapidly to the cues learned the task more slowly. For individual trials, the mean latency for responding in correct trials (*L*_C_) was significantly longer than that in incorrect trials (*L*_I_), suggesting that, on average, long deliberation times led to correct answers in the trials. The success ratio before learning was not correlated with the learning speed. Thus, deliberative decision-making, rather than overall correctness, is critical for learning.

## Introduction

Making flexible decisions is adaptive in a changing environment and constitutes intelligent behavior. Appropriate decision-making includes at least two domains: speed (quickness) and quality (correctness) [1–3]. If we spend too much time before making a decision, the decision may no longer be helpful in an ever-changing environment. Moreover, too heavy deliberation may cause inconsistent decision making [4]. On the other hand, if we decide too quickly before collecting sufficient evidence, the decision may lack precision, leading to a worse consequence. For example, in some decision-making tasks, shorter deliberation times are associated with an overall deficit in decision-making performance [5]. Therefore, quickness and correctness in decision-making are often irreconcilable. However, the relationship between the response latency and the eventual task performance has not been fully addressed under controlled experimental conditions.

The quickness-correctness relationship is also an important issue in terms of individual differences because response and learning speeds vary across individuals [6–8]. To our knowledge, no animal studies that focused on individual differences have examined this relationship. In the present study, we employed a simple task of a nose-poke behavior test in an operant chamber. The operant chamber contained two nose-poke holes, either of which was randomly illuminated by a green light in the test phase. To obtain rewards, animals were required to poke their snouts into the hole that was not illuminated. In this behavioral task, we observed various learning curves and latencies across rats, which allowed us to investigate the individual differences in behavioral parameters in decision-making.

## Methods

### Animal preparation

The experiments were performed with the approval of the animal experiment ethics committee of the University of Tokyo (approval number: 29–4) and according to the University of Tokyo guidelines for the care and use of laboratory animals. These experimental protocols were carried out in accordance with the Fundamental Guidelines for Proper Conduct of Animal Experiment and Related Activities in Academic Research Institutions (Ministry of Education, Culture, Sports, Science and Technology, Notice No. 71 of 2006), the Standards for Breeding and Housing of and Pain Alleviation for Experimental Animals (Ministry of the Environment, Notice No. 88 of 2006) and the Guidelines on the Method of Animal Disposal (Prime Minister's Office, Notice No. 40 of 1995). A total of 22 adult male Sprague Dawley rats (SLC, Shizuoka, Japan), aged 7–8 weeks when they were tested, were housed individually and maintained on a 12-h light/dark cycle (light from 6:30 to 18:30) at 22 ± 1°C with food and water available *ad libitum*. Food pellets were removed from the cages 48 h before each behavior test.

### Apparatus

An operant chamber (in mm: 300 W × 300 D × 300 H) was equipped with two nose-poke holes, two lights, a sound buzzer, and a chamber light. If a rat poked its nose into a hole in the front panel within a time limit, a food pellet (20 mg, AIN-76A Rodent Tablet; Test Diet, Richmond, IN, USA) was immediately provided from the reward port. Each trial started when the chamber light (5.5 lux, white) was turned off and ended when the rat poked its nose or when it reached the time limit. Then, the chamber light was turned on. The inter-trial intervals (ITIs) varied between 1 s and 4 s in a pseudo-random manner. One session consisted of 20 or 40 consecutive trials, and the inter-session interval was 60 s. The first and last five trials were omitted from the analyses. Rats were habituated in the chamber for 10 min on the day before the behavior test. The behavior test was composed of two phases, *i*.*e*., training and testing.

### Training and test sessions

Training sessions were performed for two consecutive days. Day 1 consisted of 3 sessions (40, 40, and 20 trials) with a time limit of 120 s, whereas Day 2 consisted of 5 sessions (40 trials each) with a time limit of 15 s. Rats were trained to poke their noses into one of two holes within these time limits. Nose-poking into either hole resulted in the delivery of a food pellet accompanied by a beep sound (200 ms, 70 dB, 3 kHz ± 0.5 kHz). Thus, either hole represented a correct choice during the training phase. Test sessions were performed on four consecutive days (Days 3–6) following the training sessions. Each day consisted of 10 sessions (40 trials in each session) with a time limit of 15 s. One of two nose-poke holes was pseudo-randomly illuminated by a green light-emitting diode in the hole (530 nm, 3.0 lux). The unilluminated hole represented the correct choice during the test phase.

### Data analyses

Data were analyzed using custom-made MATLAB routines (MathWorks, Natick, MA, USA). The rate of correct responses was calculated for each session. A correct rate of 80% was defined as the criterion for the completion of learning. The number of sessions required to achieve the criterion for the first time was used as an index of task performance. For each rat, three stages in the learning process were considered, *i*.*e*., a training period (the last four sessions on Day 2 in the training phase), a during-learning period (four sessions immediately before reaching the criterion in the test phase), and a post-learning period (four 5-to-8 sessions after reaching the criterion). The mean latencies to poking from the trial onset were measured. In some analyses, the mean latencies in correct trials (*L*_C_) and incorrect trials (*L*_I_) were separately calculated. The geometric mean of the ratios of *L*_I_ to *L*_C_ (*L*_I_/*L*_C_) in the during-learning period was used as an index that represents "carelessness" in the decision-making of a rat. Moreover, we defined the preference bias as follows:

$$Preference\;bias=|\frac{NP_{Left}-NP_{Right}}{NP_{Left}+NP_{Right}}|,$$

where NP_Left_ and NP_Right_ represent the number of pokes into the left and right holes, respectively. The preference bias ranges between 0 and 1, and a higher value indicates a greater bias to choose either of two nose-poke holes.

### Statistics

All summarized data are presented as the mean ± standard error of the mean (SEMs). To examine the statistical significance of the data, we used Tukey's test after one-way ANOVA, Pearson's correlation coefficient test, and a bootstrap test. In the bootstrap resampling method, 10,000 surrogates of the *L*_I_/*L*_C_ values for the latency comparison and CV_unilluminated_−CV_illuminated_ values for the coefficient of variation comparison (Panel D in S3 Fig) were randomly generated to determine the *P* value. The significance level was set at *P* < 0.05 for all data analyses.

## Results

**Fig 1:**
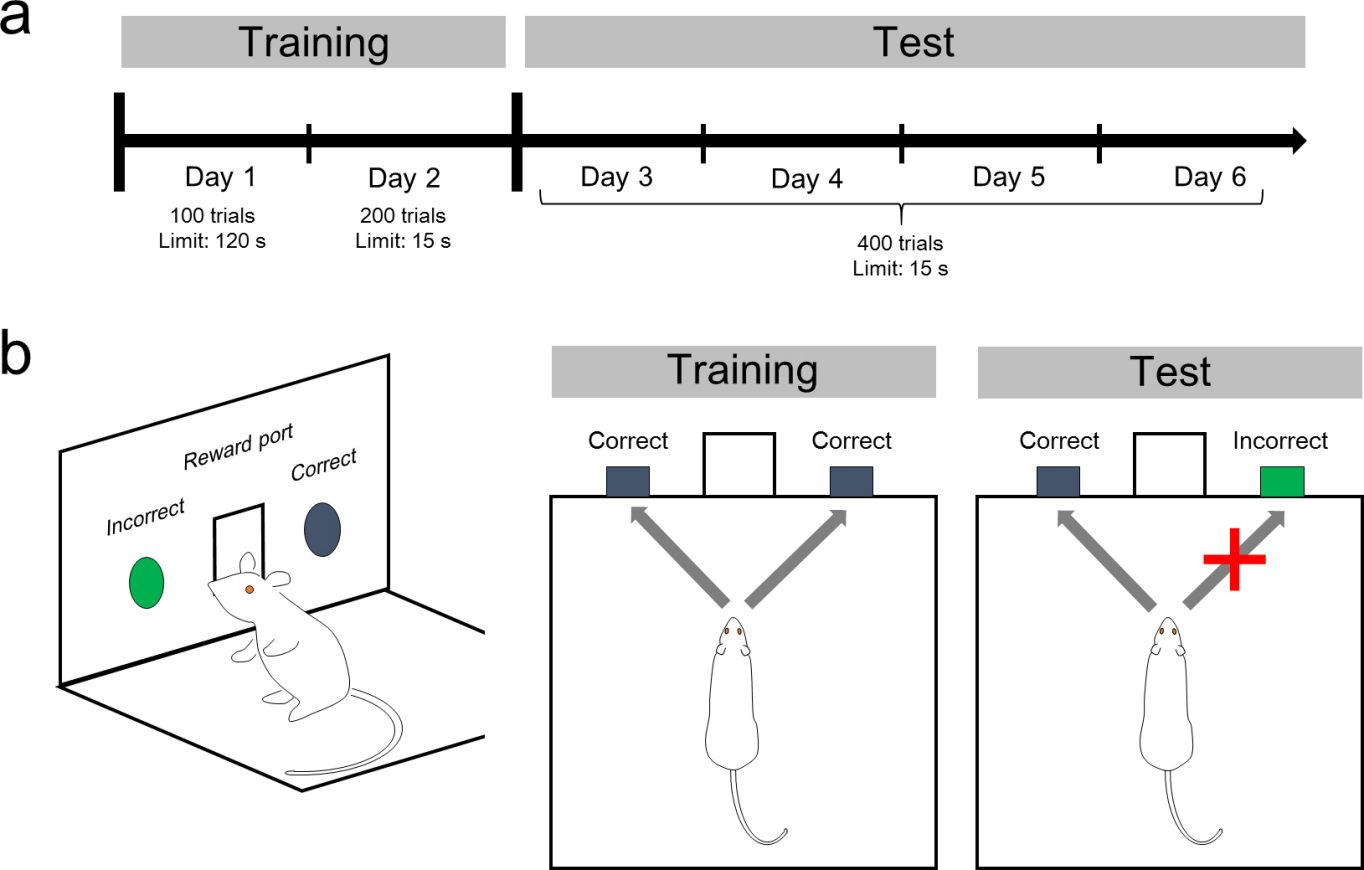
Nose-poke behavior test. (a) Experimental procedure. The behavior test consisted of a training phase (Days 1–2) and a test phase (Days 3–6). (b) Illustration of the operant chamber with the two nose-poke holes (*left*). In the training phase, a rat was rewarded whenever the nose was poked into either nose-poke hole. In the test phase, however, the rat could gain a reward pellet only when it poked the nose into the hole that was not illuminated by a green light (*right)*.

To observe the process of learning, we employed a nose-poke behavior test. This test has been used to assess the operant learning ability via gradual increases in task performance. Rats were forced to choose one of two nose-poke holes. The task consisted of two steps: a training phase and a test phase (Fig 1A). During the training phase (Days 1 and 2), both holes were correct choices; that is, rats were able to obtain food pellets when they poked their noses into either hole. The test phase that consisted of Days 3–6 began on the day following the training phase. During the test phase, a single hole was illuminated by a green light in a pseudo-random manner. Rats had to poke their snouts into the unilluminated hole (Fig 1B). If rats made a nose-poke into the illuminated hole or failed to poke within a time limit of 15 s, the trial was finished without a reward. The next trial started after an interval of 1–4 s. Success (correct response) was considered when a rat earned a reward in the trial.

Achievement of learning was considered when the mean percentage of correct trials in a given session reached a certain criterion for the first time. To optimize the criterion to determine learning completion, we tested three different criteria, i.e., 70%, 80%, and 90% correct rates (S1 Fig). We aligned the changes in the correct rates with the time when the rats reached each criterion and calculated the standard deviations (SDs) of the correct rates among animals during the during-learning period (four sessions before reaching the criterion in the test phase) and the post-learning period (four 5-to-8 sessions after reaching the criterion). The root mean square (RMS) of the SDs during these periods was the smallest for the 80% criterion (70%: RMS = 6.82, 80%: RMS = 6.11, 90%: RMS = 7.39), indicating that the criterion of the 80% correct rate was the most suitable to statistically compare the behavioral parameters. Therefore, we adopted the criterion of an 80% correct rate in the following analyses.

**Fig 2:**
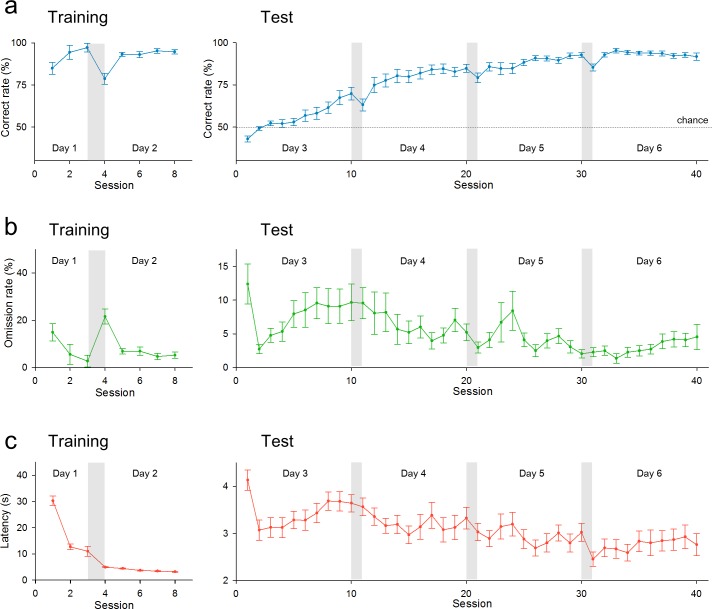
Summarized data of behavioral performance during the training and test phases of the operant task. (a) Time changes in the mean correct rates in the training phase (*left*) and the test phase (*right*). (b) Same as a, but for the mean omission rates, *i*.*e*., a percentage of trials in which the rats did not respond within the time limit. (c) Same as a, but for the mean latencies to respond. Error bars represent SEMs for 22 rats.

During the training phase, all 22 rats tested reached the criterion of learning in an early session on Day 1 (Fig 2A). The percentages of trials without poking (omission rates) and the latencies to nose-poking into the hole decreased rapidly as training progressed (Fig 2B and 2C, respectively). At the beginning of the test phase on Day 3, the percentage of correct trials decreased to approximately 50% of the chance level. However, rats gradually learned the task rule. The correct rates increased, and eventually, all rats reached the criterion within the first 3 days of the test phase (Fig 2A). The omission rates (Fig 2B) and the latencies to nose-poking (Fig 2 and Panel A in S2 Fig) were both gradually reduced during the test phase.

**Fig 3:**
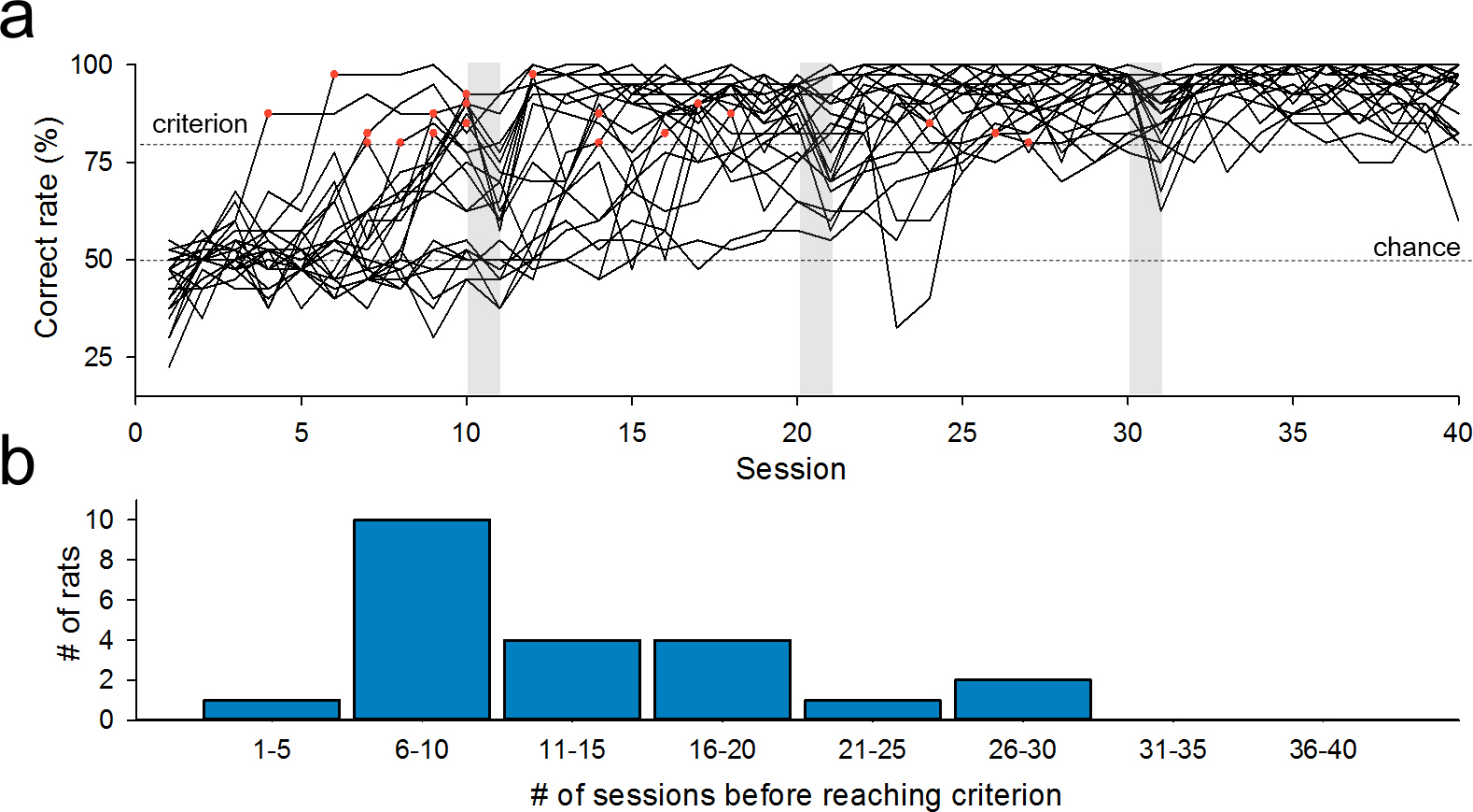
Individual differences in learning curves. (a) Time courses of the correct rates for 22 rats. Reaching the correct rate of 80% was defined as the criterion for completion of learning. Red dots indicate the first session in which the rats met the criterion. (b) Distribution of the sessions spent to reach the criterion.

**Fig 4:**
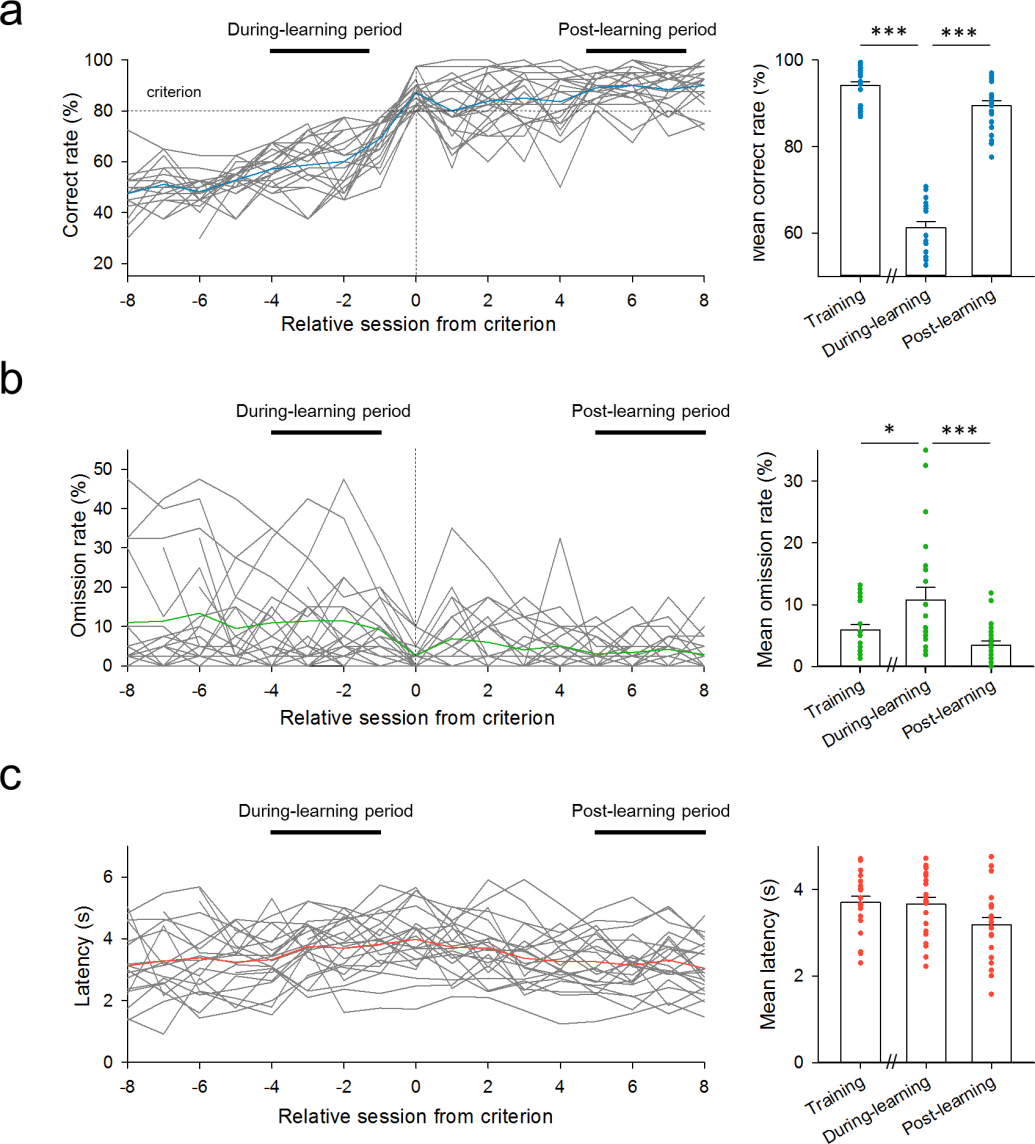
Behavioral parameters before, during, and after learning. (a) *Left*: time changes in the correct rates for 22 individual rats (gray) were aligned to the first session that reached the criterion. The blue line indicates the mean value. *Right*: training represents data from the last 4 sessions on Day 2 in the training phase. Four sessions immediately before and 5-to-8 sessions after reaching the criterion were defined as the during-learning and post-learning periods, respectively. These periods are shown by the black bars in the left panel. The mean correct rate in the during-learning period was significantly lower than the mean correct rates in the training period and the post-learning period. Training *versus* during-learning: *P =* 9.56 × 10^−10^, *Q*_3,63_ = 28.0; during-learning *versus* post-learning: *P =* 9.56 × 10^−10^, *Q*_3,63_ = 24.0; *P =* 1.14 × 10^−29^, *F*_2,63_ = 230; Tukey's test after one-way ANOVA. (b) Same as a, but for the rates of omission trials. Training *versus* during-learning: *P =* 0.034, *Q*_3,63_ = 3.62; during-learning *versus* post-learning: *P =* 6.91 × 10^−4^, *Q*_3,63_ = 5.51; *P =* 9.12 × 10^−4^, *F*_2,63_ = 7.84. (c) Same as a, but for the latencies to respond. Training *versus* during-learning: *P =* 0.984, *Q*_3,63_ = 0.235; during-learning *versus* post-learning: *P =* 0.092, *Q*_3,63_ = 3.01; *P =* 0.044, *F*_2,63_ = 3.28.

We focused on the behavioral parameters of individual rats because different rats indicated different learning curves (Fig 3A). Indeed, the number of sessions required to reach the learning criterion varied among rats, ranging from 4 to 27 sessions (Fig 3B). We aligned the time changes in the correct rates (Fig 4A, left), in the omission rates (Fig 4B, left), and in the latencies to nose-poking (Fig 4C, left) with the first sessions in which the rats reached the criterion. We then compared these behavioral parameters for three stages, *i*.*e*., the training period (the last four sessions on Day 2 in the training phase), the during-learning period, and the post-learning period (Fig 4A–4C, right). Not surprisingly, the correct rates during learning were significantly lower than those in the training and post-learning periods (Fig 4A, right; training *versus* during-learning: *P =* 9.56 × 10^−10^, *Q*_3,63_ = 28.0; during-learning *versus* post-learning period: *P =* 9.56 × 10^−10^, *Q*_3,63_ = 24.0; *P =* 1.14 × 10^−29^, *F*_2,63_ = 230; Tukey's test after one-way ANOVA). We found that the omission rates during learning were significantly higher than those in the training and post-learning periods (Fig 4B, right; training *versus* during-learning: *P =* 0.034, *Q*_3,63_ = 3.62; during-learning *versus* post-learning: *P =* 6.91 × 10^−4^, *Q*_3,63_ = 5.51; *P =* 9.12 × 10^−4^, *F*_2,63_ = 7.84). Thus, we reasoned that the change of a rule might demand that rats deliberate and adapt through trial and error. However, the latencies to poking did not differ among the three periods (Fig 4C, right; training *versus* during-learning: *P =* 0.984, *Q*_3,63_ = 0.235; during-learning *versus* post-learning: *P =* 0.092, *Q*_3,63_ = 3.01; *P =* 0.044, *F*_2,63_ = 3.28). This result suggests that rats responded steadily to the cue lights irrespective of their learning stages, but the result may also be caused by pooling data from all trials in all animals. We thus plotted the latencies only in correct trials (*L*_C_). The *L*_C_ values were significantly lower in the post-learning period than in the during-learning period (Panel B in S2 Fig; *P =* 0.0478, *Q*_3,63_ = 3.42; *P =* 0.037, *F*_2,63_ = 3.47). This reduction may represent stable choice strategies acquired by learning.

**Fig 5:**
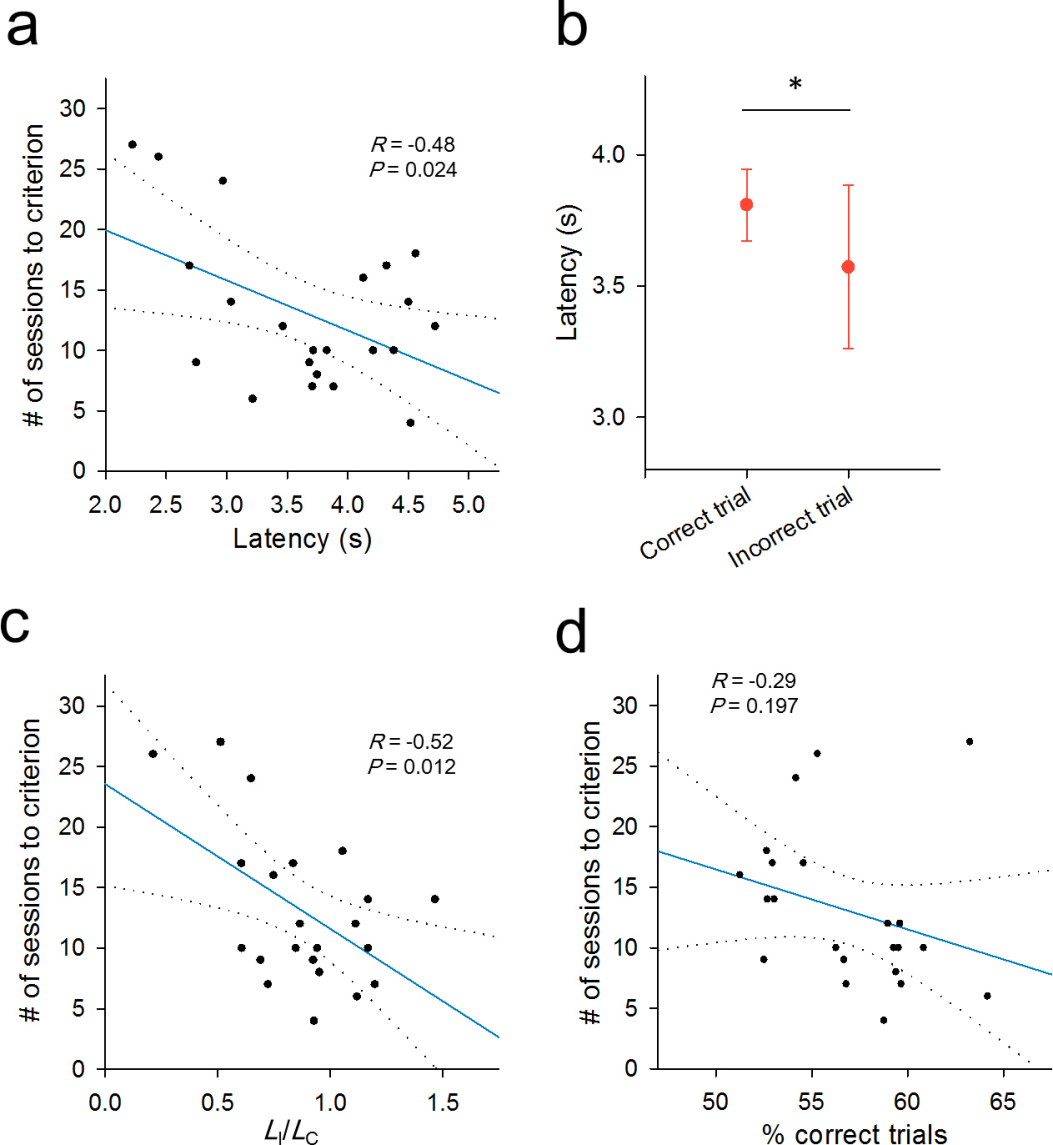
Relationship between nose-poke latencies in the during-learning period and task performance. (a) The number of sessions spent to reach the criterion is plotted against the latency to respond. Each dot indicates data from a single rat. The blue line is the best-fit line determined by the least-squares method, and its 95% confidence intervals are shown by two broken lines. As a whole, rats with shorter latencies reached the criterion more slowly (*P =* 0.024, *R* = -0.48, Pearson's correlation test, *n* = 22 rats). (b) The latencies of individual trials are separately plotted for correct trials (*L*_C_) and incorrect trials (*L*_I_). *L*_I_ was significantly shorter than *L*_C_ (*P =* 0.016, bootstrap resampling test). Error bars represent SEMs for 22 rats. (c) Same as a, but against the latency ratio *L*_I_/*L*_C_. Rats with a smaller *L*_I_/*L*_C_ ratio learned more slowly (*P =* 0.012, *R* = -0.52). (d) Same as a, but against the mean correct-trial ratio from the beginning of the test phase to the first session that reached the criterion (*P* = 0.197, *R* = -0.29).

We thus plotted the latencies for individual rats (Fig 5A). Pearson's comparison between the mean latency to poking during learning and the number of sessions until reaching the criterion revealed a significant negative correlation (*P* = 0.024, Pearson's correlation coefficient [*R*] = -0.48, *n* = 22 rats), indicating that quicker responses were associated with slower learning. We repeated the same analyses for other criteria of 70% and 90% correct rates and consistently observed similar negative correlations between the learning speed and the response latencies (Panel D-F in S1 Fig). This relationship is consistent with the idea that deliberation during learning benefits the overall task performance.

For comparisons, we also designed another behavioral test in which rats were required to poke their noses into the illuminated hole (Panel A-C in S3 Fig). In this task, we failed to find the negative correlation (*P* = 0.867, *R* = -0.078, *n* = 7 rats; Panel E in S3 Fig). Rats learned faster in the illuminated-hole-correct conditions than in the unilluminated-hole-correct conditions, and the learning speeds were no longer distributed widely across individuals (Panel D in S3 Fig); note that the coefficient of variation (CV) under the illuminated-hole-correct conditions was smaller than that under the unilluminated-hole-correct conditions (CV_illuminated_ = 0.397, CV_unilluminated_ = 0.488; *P* = 0.018, bootstrap resampling test, *n* = 7 and 22 rats, respectively). We thus speculate that the effect of deliberation varied depending on the difficulty of a given task; the negative correlations between the learning speed and the response latencies may be found only in learning tasks where the degree of difficulty is somewhat difficult.

Interestingly, the latencies to poking differed between correct and incorrect trials (Fig 5B); the mean latencies in correct trials (*L*_C_) were significantly longer than those of incorrect trials (*L*_I_) (*P* = 0.016, bootstrap resampling test, *n* = 22 rats). Thus, a thoughtless decision in each trial may lead to an undesired outcome. To compare the relationship between *L*_I_ and *L*_C_ to the task performance in each rat, we plotted the number of sessions until reaching the criterion against the ratio of *L*_I_ to *L*_C_ (Fig 5C). These parameters were also negatively correlated (*P* = 0.012, *R* = -0.52). Thus, rats that decided more quickly in error trials achieved learning more slowly.

The ratio of the number of correct trials to the total number of trials during learning was not correlated with the number of sessions before reaching the criterion (Fig 5D, *P* = 0.197, *R* = -0.29). Therefore, correctness during learning was unlikely to be critical to the overall task performance.

**Fig 6:**
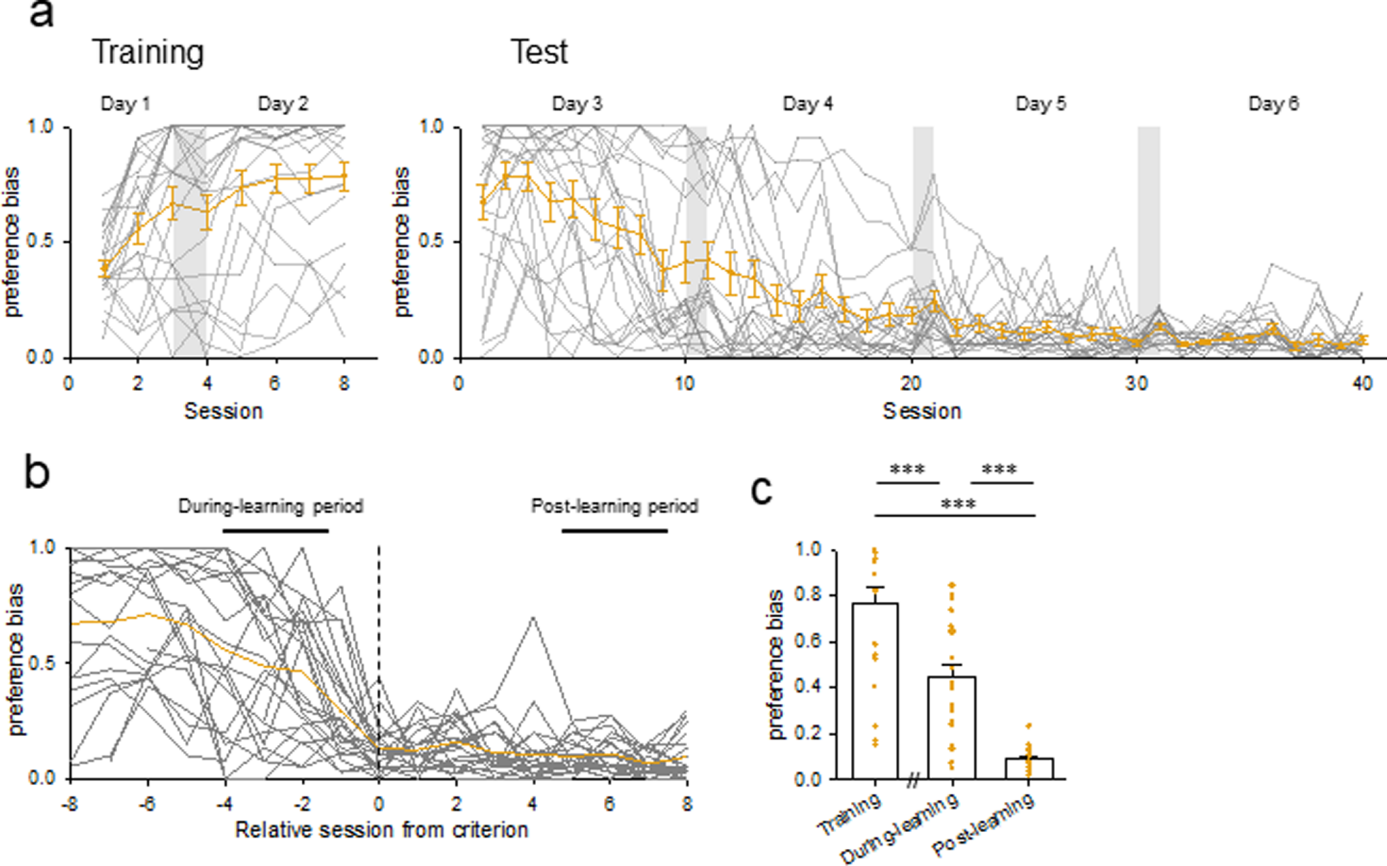
Preference bias during the training and test phases. (a) Time changes in the preference bias of individual 22 rats (gray lines) in the training phase (*left*) and the test phase (*right*). The yellow line indicates the means ± SEMs of 22 rats. (b) Same as (a), but they were aligned to the first session that reached the criterion. (c) Comparisons of the mean ± SEM preference biases during the training period, the during-learning period, and the post-learning period. The mean preference bias in the during-learning period was significantly lower than that in the training period and was significantly higher than that in the post-learning period. Training *versus* during-learning: *P =* 4.16 × 10^−5^, *Q*_3,63_ = 6.66; during-learning *versus* post-learning: *P =* 5.59 × 10^−6^, *Q*_3,63_ = 7.43; *P =* 1.12 × 10^−13^, *F*_2,63_ = 49.7; Tukey's test after one-way ANOVA.

Finally, we evaluated the cognitive paradigm tested in our behavioral task *per se*. Set-shifting tasks have often been used to examine the behavioral flexibility of animals, [9–12]. Set-shifting tasks require an extradimensional shift in attention from one stimulus dimension (such as visual-cue) to another dimension (such as direction) [9]. It was unclear that our behavioral task involves an aspect of the set-shifting tasks because the switch from the training session to the test sessions in our task did not apparently force animals to switch from poking in one hole to the other choice. To examine whether set-shift-like behavioral switches occurred in our task, we calculated the preference bias for each rat (see Methods). In the training phase, most of the rats tested showed high preference biases for holes in the training phase (Fig 6A, left). Thus, rats strategically preferred to choose one hole even though both holes were rewarded. In the test phase, their preference biases decreased to nearly zero as learning progressed (Fig 6A, right). Thus, rats switched their strategies and chose two holes more equally. Because the correct rates increased in parallel with the decrease in the preference biases, the reduced biases suggested that rats learned the rule of this task and selected the unilluminated hole to obtain rewards. Indeed, we compared the mean preference biases of the training, during-learning, and post-learning periods (Fig 6D). The preference bias in the during-learning period was significantly lower than that in the training period and that in the post-learning period was significantly lower than those in the training period and the during-learning period (Fig 6C; training *versus* during-learning: *P =* 4.16 × 10^−5^, *Q*_3,63_ = 6.66; training *versus* post-learning: P = 9.56 × 10^−10^, *Q*_3,63_ = 14.1; during-learning *versus* post-learning: *P =* 5.59 × 10^−6^, *Q*_3,63_ = 7.43; *P =* 1.12 × 10^−13^, *F*_2,63_ = 49.7). Therefore, we consider that a set-shifting-like cognitive process occurred in the test phase of our task.

## Discussion

In the present study, we analyzed rat behavioral characteristics in a nose-poke task by focusing on individual differences. Several other studies have also demonstrated individual differences in behavioral responses. For example, Olshavsky *et al*. observed cue-directed behaviors of rats [7]. In their work, rats were divided into two groups: orienters, which displayed rearing or orienting to the light cue before approaching a reward site, and nonorienters, which showed only reward-approaching behaviors. These researchers reported that orienters exhibited more impulsive and risky behaviors in a delay-discounting task and a risky decision-making task. The authors also showed that orienters failed to respond accurately when a house light blinked as a distraction, although the baseline accuracy of orienters was higher than that of non-orienters. The authors associated animals' behavioral propensities with their impulsivity or risky decision-making; however, they did not discuss individual differences in learning speed. In another study, Igata *et al*. investigated problem-solving behaviors of mice using a complex maze with multiple routes and different intersections [6]. The authors addressed the individual differences in spatial exploration and task performance and demonstrated that mice that had initially exhibited more exploratory behaviors and had taken more incorrect routes in the initial phase eventually learned the maze faster and found more flexible and appropriate solutions when parts of the maze routes were suddenly modified. However, the spatial maze used in the work was too complicated to reveal which behavioral domains produced the individual differences.

Compared with these previous studies, we adopted a simpler task procedure. In contrast to the simplicity of the task procedure *per se*, it was still difficult for the rats to grasp the task rule; thus, they exhibited variable shapes of learning curves. In other words, our task design successfully diversified the individual differences along the axis of learning speed. We mainly analyzed four parameters: correct rates, omission rates, latencies, and preference bias. The mean omission rates during learning were significantly higher than those in the training and post-learning periods, whereas the mean latencies during learning were similar to those in the training and post-learning periods. Moreover, we discovered, from an individual difference perspective, a negative correlation between the number of sessions until reaching the criterion and the response latency.

Tasks that have been commonly used to examine behavioral flexibility, such as a set-shifting task, use light cues as a correct cue [9–12]. By contrast, in our task, we presented a light cue for an incorrect hole. Because rats did not experience the light cue in the training phase during which both holes represented the correct choices, the light cue in the test phase was a novel stimulus that had a strong salience. In a previous study that used lights as a correct cue [9], habituation to the light cue during a pretraining period was conducted to reduce the salience of the stimulus. In general, this habituation enhances the task difficulty compared with tasks that have a novel light cue because, in general, rats are motivated by a novel light cue and often approach it preferentially. In our task, the light cue was linked to the incorrect hole. This mismatch linking is usually a more difficult task. Probably due to these reasons, we were able to disperse the learning speeds of individual animals. Consistent with this notion, the learning speeds in individual rats were only narrowly distributed in the task in which the illuminated hole represented a correct choice.

We found that the mean latencies until rats responded were invariant, irrespective of their learning stages. This result was unexpected because we thought that, before learning completion, rats would take longer to decide on the hole to be poked during trials, as they did not yet know the task rule. Our anticipation was based on a previous study that suggested that reaction time increases depending on the task difficulty [13]. Moreover, once learning is completed after a trial-and-error stage, the procedure may become more automated and require less effort, thereby reducing the latency [14]. In fact, the mean *L*_C_ in the post-learning period was lower than that in the during-learning period. However, the overall mean latency across correct and incorrect trials did not change depending on the learning stages, perhaps because some rats learned to poke within the 15-s time limit but did not need to hurry. Indeed, individual differences in the latencies were larger than the between-group differences. It is notable that the mean latencies in the training period were equivalent to the mean of the last 4 sessions on Day 2 in the training phase. It was probable that some rats may have already learned not to rush during the training phase whereas others may have reduced their motivation to respond rapidly because of satiety in the training period.

By contrast, the mean omission rates increased in the during-learning period compared with the training period and the post-learning period. Omission is often used as a parameter of motivation, and increased omission rates represent a less motivated state[8,10]. However, in our task, the increased rate of omitted trials during learning may not necessarily represent a decrease in motivation because the effect was only transient; omission was again reduced when learning was completed. We speculate that omissions immediately before learning completion reflect deliberation and thereby a drive to learn the task rule. In this sense, it is possible that our time limit of 15 s was too short for rats to deliberate and resulted in an increase in the omission rates.

We found a negative correlation between the number of sessions to the criterion and the response latency. From our experimental results alone, we cannot deduce the neural origin of the individual differences in learning speed and latency. Some neuromodulators may produce the individual differences. For example, dopaminergic signaling in the prefrontal cortex is reported to be essential for a set-shifting task [10,15]. More specifically, the local infusion of antagonists for dopamine D_2_ and D_4_ receptors into the prefrontal cortex impaired the performance of a maze-based set-shifting task, whereas the infusion of a dopamine D_4_ antagonist improved it. Other studies have demonstrated the involvement of serotonin in an attentional set-shifting task[16,17], which measures the ability of animals to switch their attention to a new stimulus that they have not learned. Performance on this type of task is facilitated by the pharmacological blockade of serotonin 5-HT_7_ receptors or the activation of serotonin 5-HT_6_ receptors[16,17].

We analyzed the preference bias in the training and test phase and found that the preference bias that had been high in the training phase decreased gradually in the test phase as learning proceeded. The high preference biases in the training period might reflect automated responses [14]. Indeed, in the first session in the test phase, the preference bias was still high but soon started to decrease. Therefore, set-shifting-like changes in the strategies taken by rats were likely to occur during learning.

A shorter *L*_I_ was also reported in a discrimination task of the motion direction in coherent random dot stimuli [13]. In another study, however, the latency did not differ between rewarded responses and non-rewarded responses [8]. This inconsistency may be due to the difference in the task difficulty or the difference in the definition of learning periods or stages. Moreover, we compared *L*_I_ and *L*_C_ in the during-learning period; note that, to our knowledge, no previous studies have analyzed the latency in the dimensions of task success and failure. We found that quicker responses were associated with more errors. Furthermore, we showed that lower *L*_I_/*L*_C_ ratios were associated with more sessions before learning completion. Therefore, rats that made faster decisions, especially in error trials, eventually exhibited worse task performance. It is also notable that we introduced a new index, the *L*_I_/*L*_C_ ratio. Because the index can extract the internal difference between correct and incorrect responses within an animal, we believe that the *L*_I_/*L*_C_ ratio reflects the animal’s internal states that generate strategies and is applicable to analyze the psychological states of other species including human’s studies.

## Supporting information

S1 Fig Effects of different criteria for learning completion. (a-c) Time changes in the correct rates in 22 individual rats (gray) were aligned to the first session that reached 70% (a), 80% (b), and 90% (c) correct rates. The colored lines indicate the mean values. Note that data of the criterion of an 80% correct rate are identical to Fig 4A. (d-f) The numbers of sessions spent to reach the criterion of 70% (d), 80% (e), and 90% (f) are plotted against the mean latencies to respond. Each dot indicates a single rat. The colored lines are the best-fit lines determined by the least-squares method, and its 95% confidential intervals are shown by two broken lines.
(TIF)

S2 Fig Nose-poke latencies in correct trials. (a) Time changes in the mean latencies in correct trials (*L*_C_) in the test phase. (b) *Left*: Same as Fig 4C, but for the latencies to respond in correct trials. The brown line indicates the mean value. *Right*: The mean *L*_C_ in the post-learning period was significantly lower than that in the during-learning period (*P =* 0.0478, *Q*_3,63_ = 3.42; *P =* 0.037, *F*_2,63_ = 3.47; Tukey's test after one-way ANOVA). Error bars represent SEMs for 22 rats.
(TIF)

S3 Fig Nose-poke behavior tests to choose the illuminate hole. (a) Task conditions in the test phase. The training phase was the same as the original procedure described in Fig 1B, but the conditions in the test phase were different from the original test. Rats gained food pellets only when they poked their noses into the illuminated hole. (b) Time courses of the correct rates for 7 rats. Reaching the correct rate of 80% was defined as the criterion for completion of learning. Red dots indicate the first session in which the rats met the criterion. (c) Distribution of the sessions spent to reach the criterion. (d) Comparison of the number of sessions to spent to reach the criterion in two tasks in which poking into the illuminated hole is a correct response (illuminated condition, *Left*) and in which the unilluminated hole is a correct one (the unilluminated condition, *Right*). The coefficient of variation (CV) of the illuminated condition is smaller than that of the original one. Error bars represent SDs for 7 rats (illuminated condition) and 22 rats (unilluminated condition). *P* = 0.018, bootstrap resampling test. (e) The numbers of sessions to spent to reach the criterion are plotted against the latencies to respond (*P* = 0.867, *R* = -0.078).
(TIF)

## References

[1] Schouten JF, Bekker JA (1967) Reaction time and accuracy. Acta Psychol (Amst) 27: 143–153. 6062205 10.1016/0001-6918(67)90054-6

[2] Bogacz R, Wagenmakers EJ, Forstmann BU, Nieuwenhuis S (2010) The neural basis of the speed-accuracy tradeoff. Trends Neurosci 33: 10–16. doi: 10.1016/j.tins.2009.09.002 19819033 10.1016/j.tins.2009.09.002

[3] Heitz RP, Schall JD (2012) Neural mechanisms of speed-accuracy tradeoff. Neuron 76: 616–628. doi: 10.1016/j.neuron.2012.08.030 23141072 10.1016/j.neuron.2012.08.030 PMC3576837

[4] Nordgren LF, Dijksterhuis AP (2009) The devil is in the deliberation: Thinking too much reduces preference consistency. J Consum Res 1: 39–46.

[5] Franken IH, van Strien JW, Nijs I, Muris P (2008) Impulsivity is associated with behavioral decision-making deficits. Psychiatry Res 158: 155–163. doi: 10.1016/j.psychres.2007.06.002 18215765 10.1016/j.psychres.2007.06.002

[6] Igata H, Sasaki T, Ikegaya Y (2016) Early Failures Benefit Subsequent Task Performance. Sci Rep 6: 21293 doi: 10.1038/srep21293 26883387 10.1038/srep21293 PMC4756702

[7] Olshavsky ME, Shumake J, Rosenthal AA, Kaddour-Djebbar A, Gonzalez-Lima F, Setlow B, et al (2014) Impulsivity, risk-taking, and distractibility in rats exhibiting robust conditioned orienting behaviors. J Exp Anal Behav 102: 162–178. doi: 10.1002/jeab.104 25130520 10.1002/jeab.104

[8] Klanker M, Sandberg T, Joosten R, Willuhn I, Feenstra M, Denys D (2015) Phasic dopamine release induced by positive feedback predicts individual differences in reversal learning. Neurobiol Learn Mem 125: 135–145. doi: 10.1016/j.nlm.2015.08.011 26343836 10.1016/j.nlm.2015.08.011 PMC5650052

[9] Floresco SB, Block AE, Tse MT (2008) Inactivation of the medial prefrontal cortex of the rat impairs strategy set-shifting, but not reversal learning, using a novel, automated procedure. Behav Brain Res 190: 85–96. doi: 10.1016/j.bbr.2008.02.008 18359099 10.1016/j.bbr.2008.02.008

[10] Butts KA, Floresco SB, Phillips AG (2013) Acute stress impairs set-shifting but not reversal learning. Behav Brain Res 252: 222–229. doi: 10.1016/j.bbr.2013.06.007 23764458 10.1016/j.bbr.2013.06.007

[11] Thai CA, Zhang Y, Howland JG (2013) Effects of acute restraint stress on set-shifting and reversal learning in male rats. Cogn Affect Behav Neurosci 13: 164–173. doi: 10.3758/s13415-012-0124-8 23055093 10.3758/s13415-012-0124-8 PMC4457521

[12] Iguchi Y, Kosugi S, Lin Z, Nishikawa H, Minabe Y, Toda S (2015) Pre-stress performance in an instrumental training predicts post-stress behavioral alterations in chronically stressed rats. Front Behav Neurosci 9: 119 doi: 10.3389/fnbeh.2015.00119 26029067 10.3389/fnbeh.2015.00119 PMC4429589

[13] Reinagel P (2013) Speed and accuracy of visual motion discrimination by rats. PLoS One 8: e68505 doi: 10.1371/journal.pone.0068505 23840856 10.1371/journal.pone.0068505 PMC3695925

[14] Redish AD (2016) Vicarious trial and error. Nat Rev Neurosci 17: 147–159. doi: 10.1038/nrn.2015.30 26891625 10.1038/nrn.2015.30 PMC5029271

[15] Floresco SB, Magyar O, Ghods-Sharifi S, Vexelman C, Tse MT (2006) Multiple dopamine receptor subtypes in the medial prefrontal cortex of the rat regulate set-shifting. Neuropsychopharmacology 31: 297–309. doi: 10.1038/sj.npp.1300825 16012531 10.1038/sj.npp.1300825

[16] Nikiforuk A (2012) Selective blockade of 5-HT7 receptors facilitates attentional set-shifting in stressed and control rats. Behav Brain Res 226: 118–123. doi: 10.1016/j.bbr.2011.09.006 21925545 10.1016/j.bbr.2011.09.006

[17] Burnham KE, Baxter MG, Bainton JR, Southam E, Dawson LA, Bannerman DM, et al (2010) Activation of 5-HT(6) receptors facilitates attentional set shifting. Psychopharmacology (Berl) 208: 13–21. 19902184 10.1007/s00213-009-1701-6

