# Supplementary figures and images for: Answering hastily retards learning
Source: PLoS One. 2018 Apr 25;13(4):e0195404. doi: 10.1371/journal.pone.0195404 (PMC5918621; doi:10.1371/journal.pone.0195404)

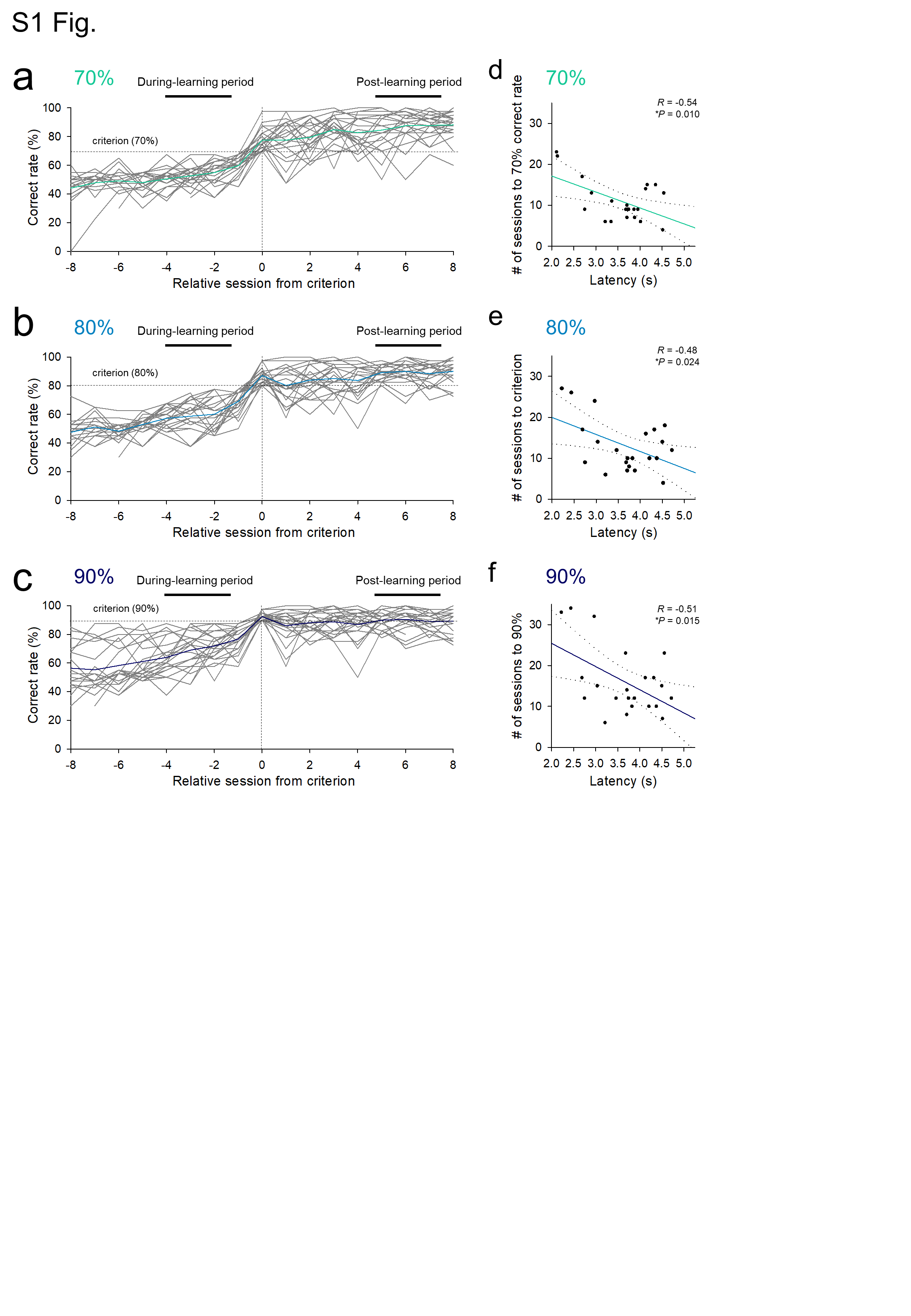

Supplement: S1 Fig — (a-c) Time changes in the correct rates in 22 individual rats (gray) were aligned to the first session that reached 70% (a), 80% (b), and 90% (c) correct rates. The colored lines indicate the mean values. Note that data of the criterion of an 80% correct rate are identical to Fig 4A. (d-f) The numbers of sessions spent to reach the criterion of 70% (d), 80% (e), and 90% (f) are plotted against the mean latencies to respond. Each dot indicates a single rat. The colored lines are the best-fit lines determined by the least-squares method, and its 95% confidential intervals are shown by two broken lines. (TIF) [file pone.0195404.s001.TIF]

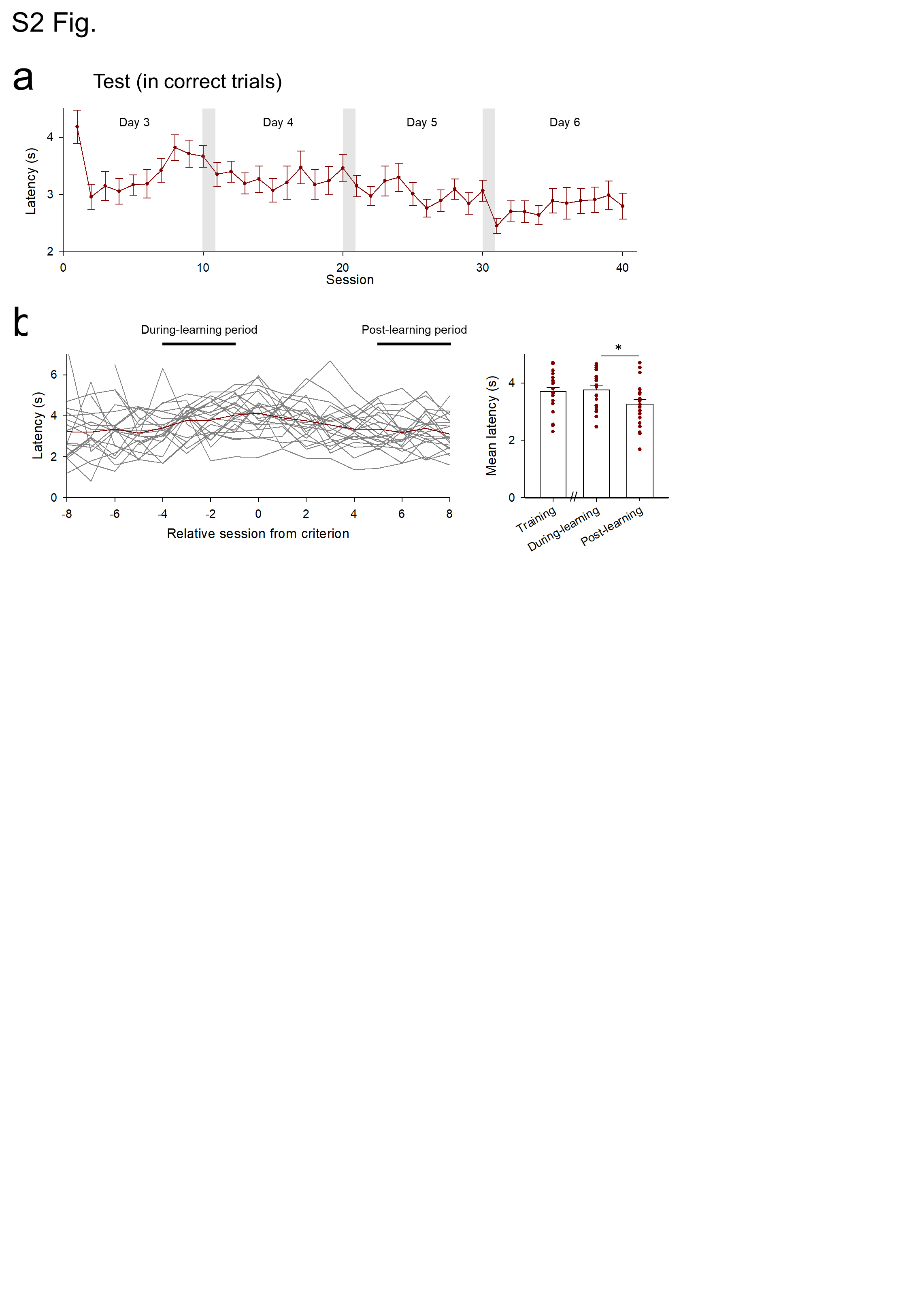

Supplement: S2 Fig — (a) Time changes in the mean latencies in correct trials (LC) in the test phase. (b) Left: Same as Fig 4C, but for the latencies to respond in correct trials. The brown line indicates the mean value. Right: The mean LC in the post-learning period was significantly lower than that in the during-learning period (P = 0.0478, Q3,63 = 3.42; P = 0.037, F2,63 = 3.47; Tukey's test after one-way ANOVA). Error bars represent SEMs for 22 rats. (TIF) [file pone.0195404.s002.TIF]

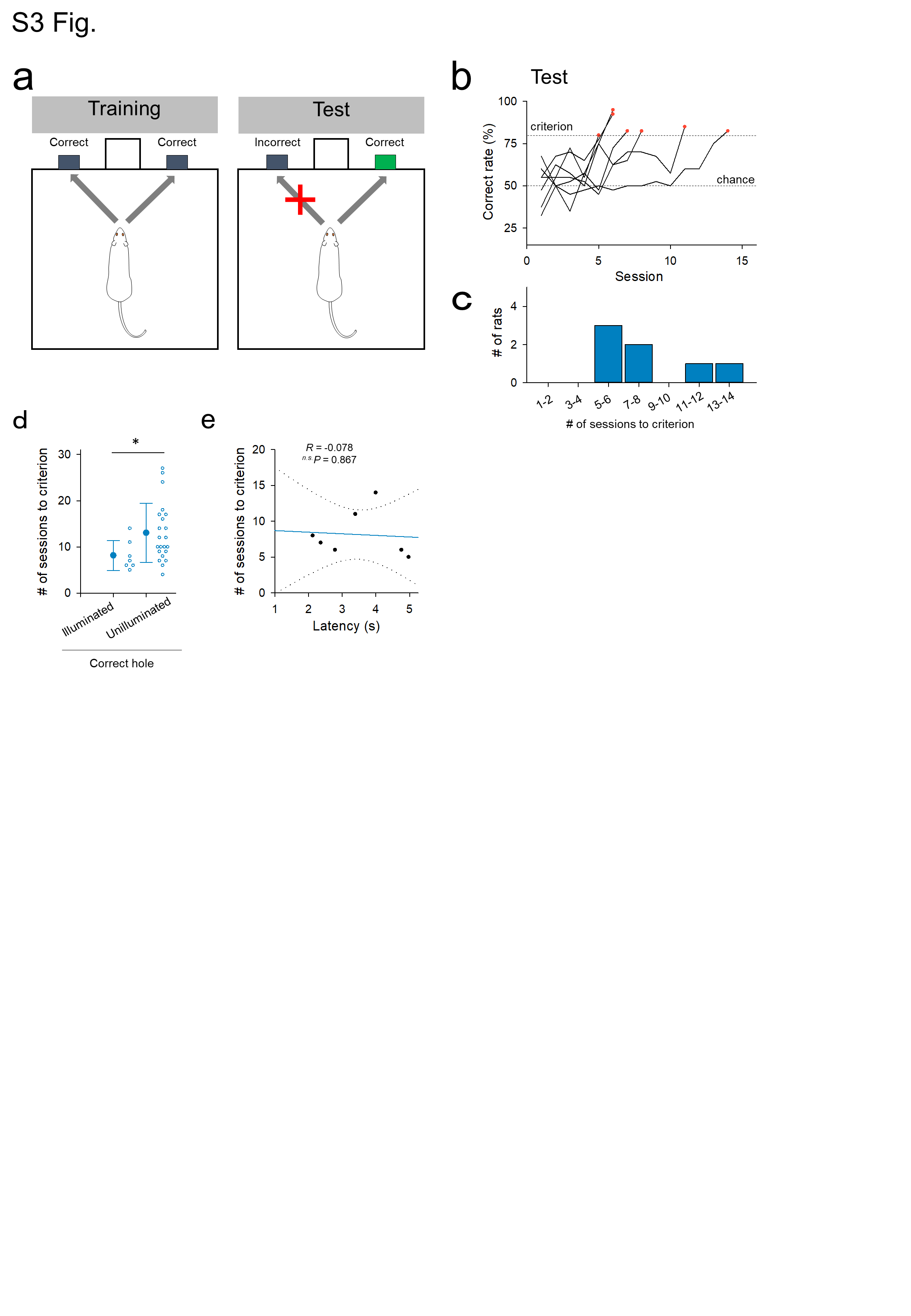

Supplement: S3 Fig — (a) Task conditions in the test phase. The training phase was the same as the original procedure described in Fig 1B, but the conditions in the test phase were different from the original test. Rats gained food pellets only when they poked their noses into the illuminated hole. (b) Time courses of the correct rates for 7 rats. Reaching the correct rate of 80% was defined as the criterion for completion of learning. Red dots indicate the first session in which the rats met the criterion. (c) Distribution of the sessions spent to reach the criterion. (d) Comparison of the number of sessions to spent to reach the criterion in two tasks in which poking into the illuminated hole is a correct response (illuminated condition, Left) and in which the unilluminated hole is a correct one (the unilluminated condition, Right). The coefficient of variation (CV) of the illuminated condition is smaller than that of the original one. Error bars represent SDs for 7 rats (illuminated condition) and 22 rats (unilluminated condition). P = 0.018, bootstrap resampling test. (e) The numbers of sessions to spent to reach the criterion are plotted against the latencies to respond (P = 0.867, R = -0.078). (TIF) [file pone.0195404.s003.TIF]
